# Frontiers and emerging trends in research on metabolic syndrome and metabolic dysfunction-associated fatty liver disease: a bibliometric analysis (2005-2024)

**DOI:** 10.3389/fendo.2026.1748699

**Published:** 2026-05-22

**Authors:** Weisong Zhang, Guangchen Liu, Xue Qu, Delong Cong, Yangyang Liu

**Affiliations:** College of Traditional Chinese Medicine, Changchun University of Chinese Medicine, Changchun, Jilin, China

**Keywords:** bibliometric analysis, citespace, hotspots, metabolic dysfunction-associated fatty liver disease, metabolic syndrome, VOSviewer

## Abstract

**Objective:**

This study aims to elucidate current trends and research hotspots in the field of metabolic syndrome (MetS) and metabolic dysfunction-associated fatty liver disease (MAFLD), thereby offering fresh perspectives to guide future investigations.

**Methods:**

A comprehensive search was conducted in the Web of Science Core Collection (WoSCC) and Scopus databases to identify relevant publications on MetS and MAFLD published between 2005 and 2024. Bibliometric analysis was performed using VOSviewer (version 1.6.20) and CiteSpace (version 6.4.R1) to systematically evaluate multidimensional metrics, including the contributions of countries/regions, institutions, journals, and authors. Furthermore, collaborative networks were constructed and keyword distribution patterns were analyzed.

**Results:**

This study incorporated a total of 9,821 publications from 120 countries/regions. Scientific output demonstrated a substantial increase from 2005 to 2024. The United States led with 2,426 publications, followed by China and Italy. The University of California System was the most prolific research institution, and the *World Journal of Gastroenterology* was the leading journal in terms of publication volume. Targher Giovanni emerged as the most influential contributor with the highest centrality, followed by Younossi Zobair M. and Yilmaz Yusuf. Analysis of keyword trends revealed an evolution from early focuses on pathophysiological foundations such as “insulin resistance” and “morbid obesity” to emerging terminology including “mitochondrial dysfunction” and “controlled attenuation parameter”. In recent years, research frontiers have advanced towards “gut microbiota”, the “gut-liver axis”, and lifestyle interventions. This evolutionary trajectory signifies a profound paradigm shift within the field, moving from traditional pathophysiological description towards mechanism-based precise diagnosis and early intervention strategies.

## 1 Introduction

Metabolic syndrome (MetS) is defined as a cluster of interrelated conditions, including central obesity, hypertension, dyslipidemia, insulin resistance (IR), and impaired glucose tolerance (1). The presence of these factors collectively contribute to a significantly elevated risk of developing type 2 diabetes mellitus (T2DM), cardiovascular diseases (CVD), and stroke (2, 3). The global prevalence of MetS is rising rapidly, affecting approximately one-quarter of the world’s population (4). According to estimates from the Centers for Disease Control and Prevention (CDC), the prevalence of MetS is 34.7% in the United States (5) and 33.9% in China (6). MetS exhibits a significant comorbidity with non-alcoholic fatty liver disease (NAFLD), as these two conditions interact through shared pathophysiological mechanisms, posing a major challenge in the diagnosis and management of metabolic disorders today (7).

Non-alcoholic fatty liver disease (NAFLD) represents the most prevalent cause of chronic liver disease worldwide and is characterized by excessive lipid accumulation in the liver (8). Its global prevalence in adults ranges from 6% to 35% (9), with a higher incidence observed in individuals with obesity, cardiovascular disease (CVD), type 2 diabetes mellitus (T2DM), and dyslipidemia (10, 11). Furthermore, NAFLD has emerged as a leading cause of cirrhosis in some countries (12). Moreover, studies have identified associations between NAFLD and various endocrine and metabolic disorders, including polycystic ovary syndrome (PCOS), hypothyroidism, hypogonadism, and growth hormone deficiency (13). With the deepening understanding of NAFLD, it is increasingly recognized that NAFLD is not merely a localized liver disorder but may also constitute a significant manifestation and driving factor of systemic metabolic dysregulation.

NAFLD is closely associated with MetS. The increasing prevalence of obesity and T2DM has further elevated the overall incidence of both MetS and NAFLD (14). Indeed, NAFLD is often regarded as the hepatic manifestation of MetS (15–17). Numerous epidemiological studies have demonstrated a robust association between NAFLD and MetS (18–20). A pivotal change was introduced in 2020 by an international expert consensus, which proposed a significant revision to the diagnostic criteria for NAFLD, advocating for the adoption of new nomenclature: metabolic dysfunction-associated fatty liver disease (MAFLD) and metabolic dysfunction-associated steatohepatitis (MASH) (21). This transformative diagnostic framework underscores the central role of metabolic dysfunction in the disease. It represents a milestone in metabolic disease research and further reinforces the intrinsic link between MAFLD and the individual components of MetS.

To facilitate the early identification of high-risk factors and halt the progression of both MAFLD and MetS in clinical practice, this review synthesizes two decades of research. Utilizing bibliometric analysis, we systematically map the associations between MAFLD and MetS, with a focus on presenting pivotal findings and emerging trends. This work aims to offer novel perspectives for the future management of these intertwined conditions.

## 2 Research methods

### 2.1 Data collection

**Figure 1:**
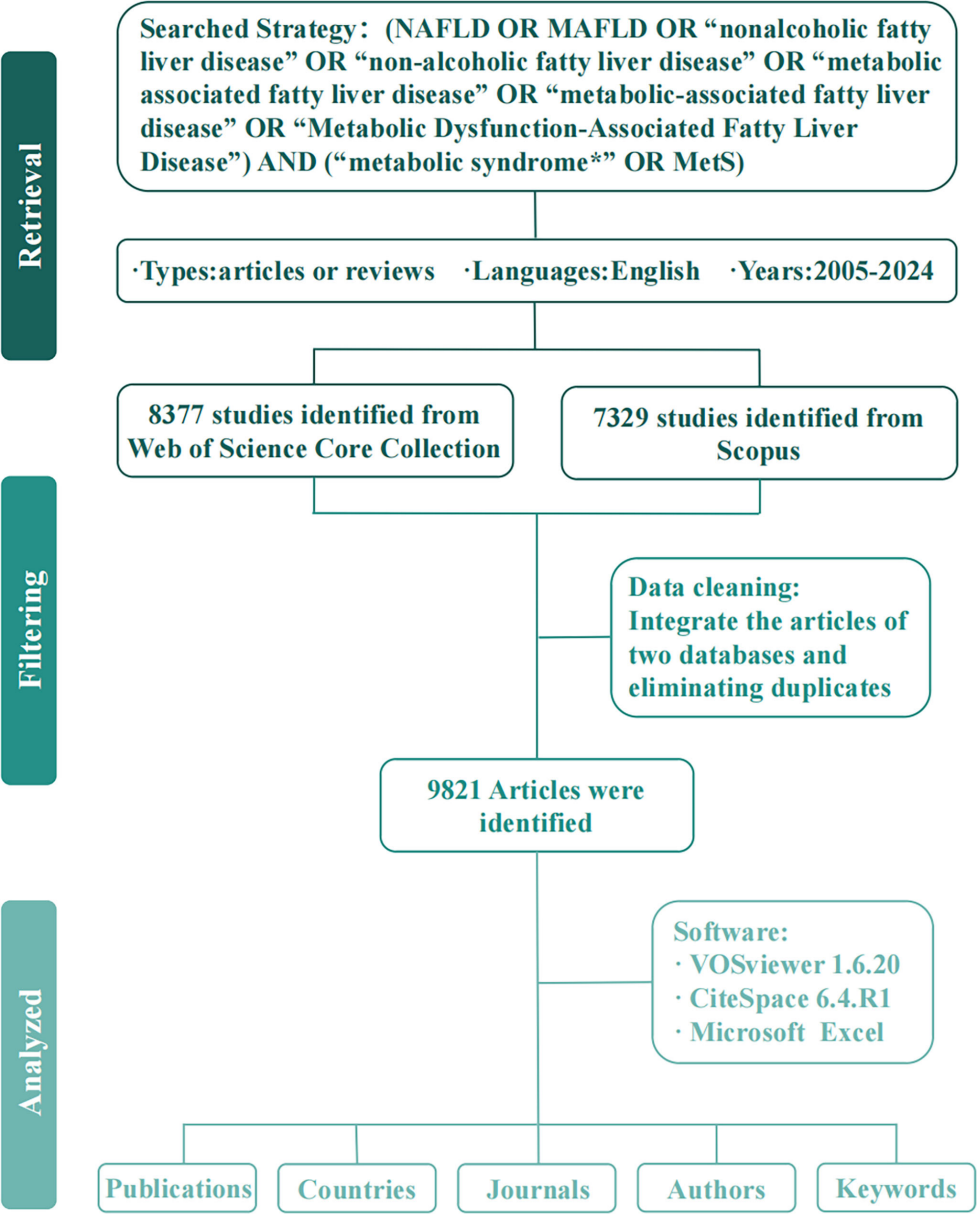
Flowchart of literature screening.

The Web of Science Core Collection (WoSCC) and Scopus databases were selected to provide comprehensive data support for this bibliometric analysis. WoSCC was chosen for its extensive coverage of high-quality medical research across disciplines such as morphology, immunology, and chemotherapy. Specifically, its indexing services—including the Science Citation Index Expanded (SCIE), Social Sciences Citation Index (SSCI), Arts & Humanities Citation Index (A&HCI), and Emerging Sources Citation Index (ESCI)—ensure access to authoritative publications on MetS and MAFLD. Scopus was additionally utilized as it represents the largest abstract and citation database of peer-reviewed interdisciplinary literature. Therefore, this study used WoSCC and Scopus as data sources. The search was conducted on November 4, 2025, and both databases were searched on the same day. The search strategy was formulated as follows: “[TS=(NAFLD OR MAFLD OR “nonalcoholic fatty liver disease” OR “non-alcoholic fatty liver disease” OR “metabolic associated fatty liver disease” OR “metabolic-associated fatty liver disease”) AND (“metabolic syndrome*” OR MetS)]”, where TS denotes a topic search. The asterisk (*) represents fuzzy searching to match any possible character combinations, while phrases in double quotation marks (“) are taken as exact matches. The document types were restricted to “Article” and “Review Article” to ensure data quality. Review articles were included to synthesize high-quality evidence, identify research gaps, and propose potential solutions. The search was limited to publications in English. This effort initially yielded 15706 records in total (8377 from WOSCC, and 7329 from Scopus). The literature information was downloaded in plain text and CSV files, encompassing complete details such as the title, authors, institutions, country, publication year, abstract, keywords, and references. The CSV files from Scopus were converted into a structure compatible with the WoSCC plain text format using Python (version 3.11). This process mainly consisted of six steps: field mapping, multi-line field reconstruction, reference parsing and structural reconstruction, author and affiliation matching, affiliation filtering and standardization, and finally exporting in the plain text format. Subsequently, data cleaning was performed using Python (version 3.11), which included removing duplicate records based on Digital Object Identifier (DOI); for records without a DOI, further deduplication was conducted by matching titles, authors, journal names, and publication years; rows containing “[Anonymous]” were deleted; virtual institutions such as the “Egyptian Knowledge Bank (EKB)” were removed; and duplicate institutions were also eliminated. All potential duplicate records were manually reviewed to ensure the accuracy of the deduplication process. Ultimately, 9821 records were selected for examination (**Figure 1**).

### 2.2 Bibliometric and visualization analysis

This study employed two mainstream bibliometric tools for a comprehensive analysis. VOSviewer (version 1.6.20) (22), a software tool for constructing and visualizing bibliometric networks developed by van Eck and Waltman (23, 24), was utilized. Using VOSviewer, we performed analyses of international collaboration networks, journal co-citation networks, and bibliographic coupling (25). The relationships between research items are represented by nodes and links within these networks. When constructing the above networks, the full counting method was adopted to build collaboration and co-occurrence networks (22), and the normalization method was set to VOSviewer’s default association strength algorithm (26).

CiteSpace (version 6.4.R1 Advanced) (27), a highly recognized tool in bibliometrics (28–30), was applied to visualize the landscape of the knowledge domain and to identify research hotspots, trends, and frontiers through its dynamic network analysis capabilities. Specifically, we used it for keyword clustering and timeline analysis. All statistical analyses and generation of figures were conducted using Microsoft Office Excel 2019.

## 3 Result

### 3.1 Quantitative analysis of publications

**Figure 2:**
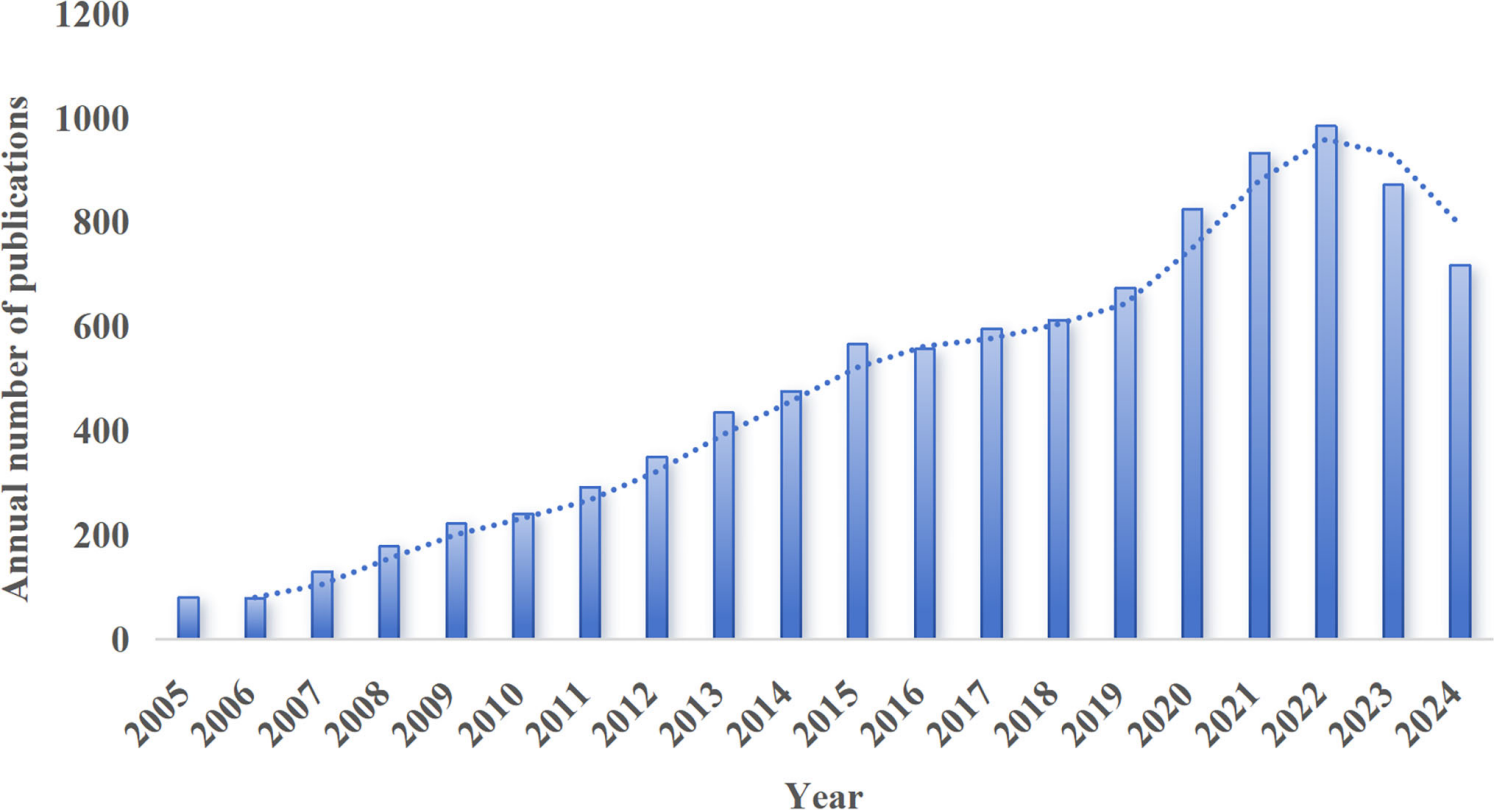
Annual publication trend of MetS and MAFLD related literature.

Based on our search strategy, a total of 9,821 publications focusing on the association between MetS and MAFLD have been identified since 2005. The annual publication output demonstrated a consistent upward trend, with a notable surge observed during the 2020–2022 period (**Figure 2**). Over the past two decades, these 9,821 publications were contributed by 44,299 authors from 5,627 institutions across 120 countries and were disseminated through 1,872 distinct scientific journals. These metrics underscore the enduring relevance and significant academic impact of research in this field.

### 3.2 Analysis of countries and regions

**Figure 3:**
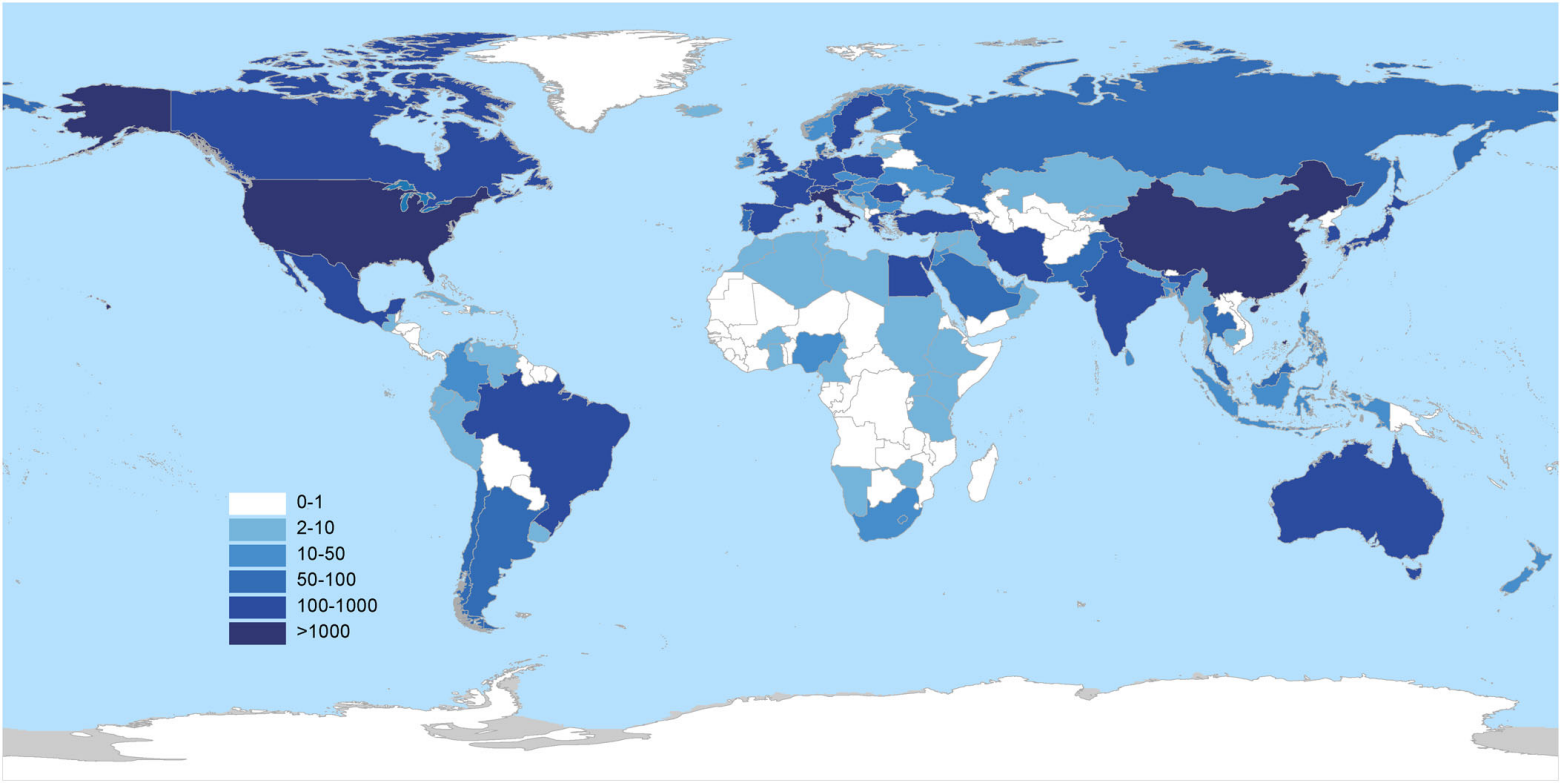
World map of research contributions based on publication count.

**Figure 4:**
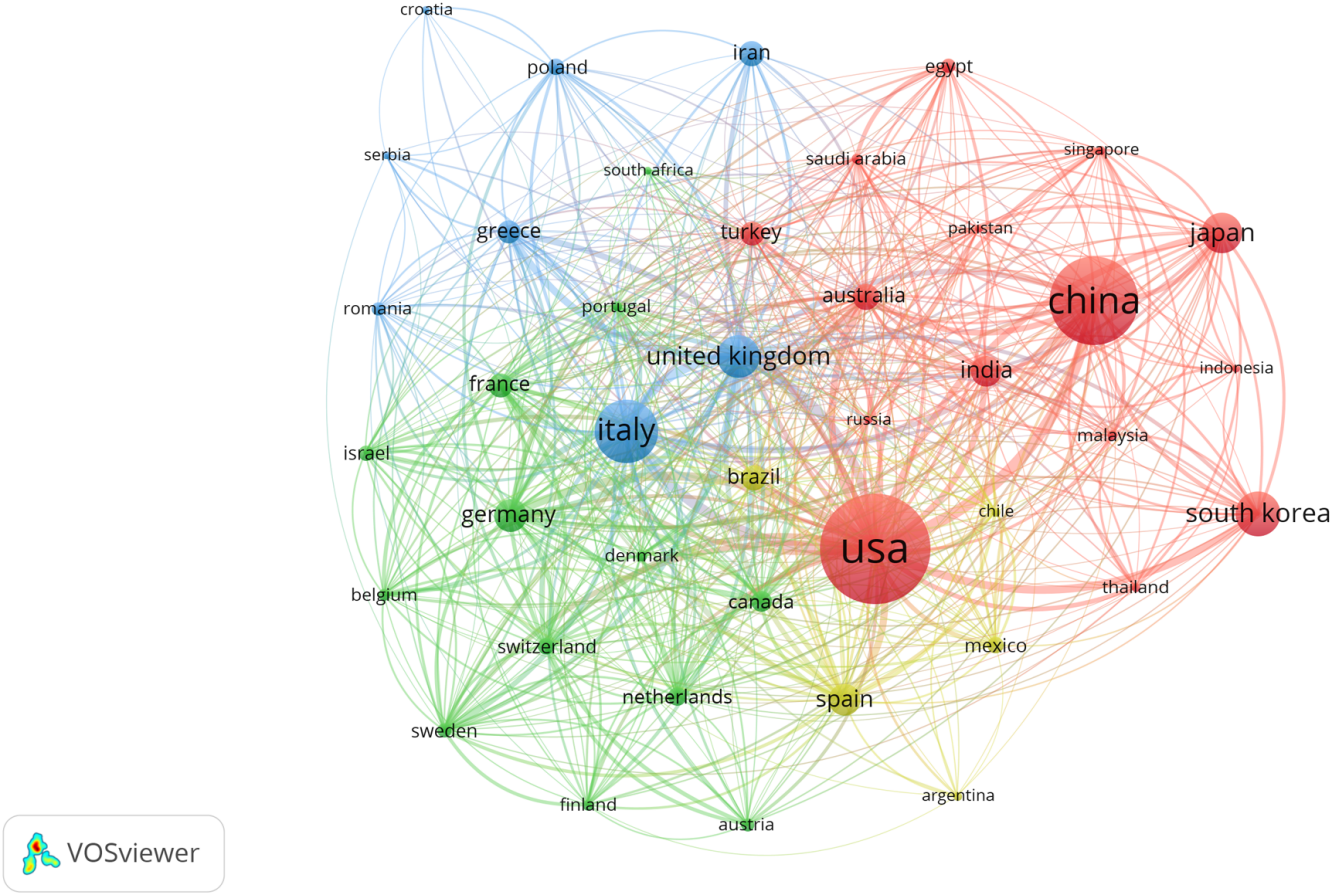
International collaboration network.

The identified publications originated from 120 countries. The United States led in publication output with 2,426 articles, accounting for 24.7% of the total. Other leading contributing countries included China (1,765 publications), Italy (1,072), South Korea (644), the United Kingdom (616), Japan (544), and Germany (410) (**Table 1**). To better understand the global distribution and collaborative landscape, the contributions of countries in this research field were visualized based on their respective publication counts (**Figure 3**). Using the Co-authorship-Countries function in VOSviewer with a minimum publication threshold of 30 articles per country, 41 countries that had published more than 30 papers on the topic were identified. Visualization of the international collaboration network revealed that the United States, China, and Italy formed the most robust and interconnected collaborative cluster (**Figure 4**).

**Table 1:** Profile of leading publishing countries.

Country	Documents	Citations	Total link strength	Average citations
USA	2426	176588	1511	72.79
China	1765	60812	622	34.45
Italy	1072	77574	758	72.36
South Korea	644	23546	230	36.56
United Kingdom	616	49849	778	80.92
Japan	554	25866	266	46.69
Germany	410	22767	568	55.53
Spain	399	16285	420	40.81
India	384	8467	208	22.05
Australia	298	28077	373	94.22

### 3.3 Analysis of institutions

**Figure 5:**
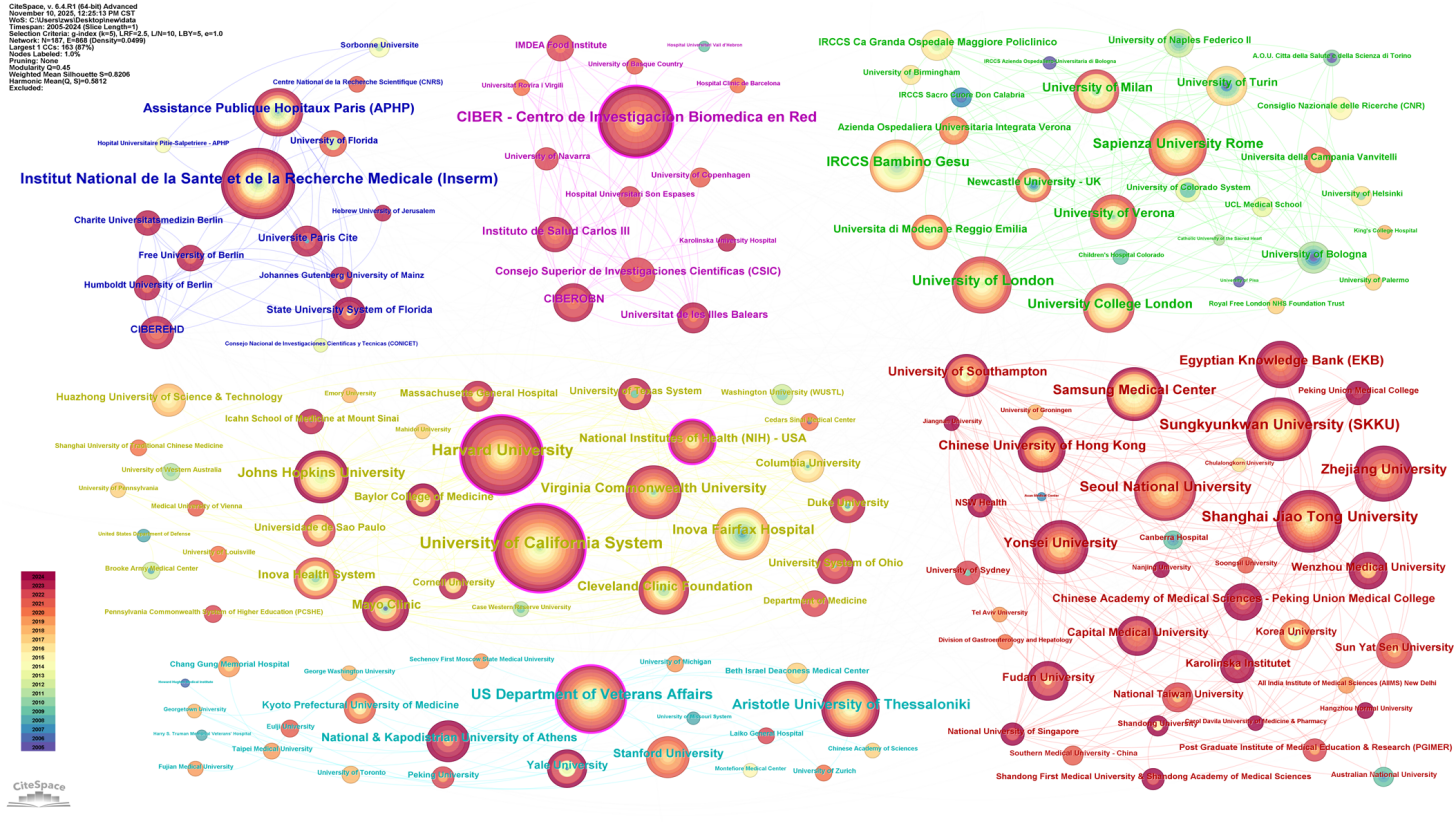
Inter-institutional collaboration network.

The 9,821 publications on MetS and MAFLD involved 5,627 research institutions. An institutional collaboration network was constructed in CiteSpace using a g-index (k=5) for node selection, where connecting lines represent collaborative relationships between institutions (**Figure 5**). The University of California System, represented by the largest node, demonstrates its extensive collaboration with other institutions. The most prolific institutions were, in descending order: the University of California System (USA, 243 publications), Harvard University (USA, 169 publications), and INSERM (France, 137 publications). The institutions with the highest centrality were: Harvard University (USA, 0.2), the US Department of Veterans Affairs (USA, 0.18), and the University of California System (USA, 0.17). The centrality indicator reflects the connection strength and mediating role of nodes within the network, indicating that the aforementioned institutions form the core of the entire network and play a crucial hub role in the flow of knowledge (**Table 2**).

**Table 2:** Profile of leading publishing institutions.

Organization	Documents	Centrality
University of California System	243	0.17
Harvard University	169	0.20
Institut National de la Sante et de la Recherche Medicale (INSERM)	137	0.05
CIBER - Centro de Investigacion Biomedica en Red	128	0.15
US Department of Veterans Affairs	125	0.18
Sungkyunkwan University (SKKU)	117	0.04
Shanghai Jiao Tong University	115	0.08
Aristotle University of Thessaloniki	101	0.01
Seoul National University	101	0.01
University of London	95	0.05

### 3.4 Analysis of journals

**Figure 6:**
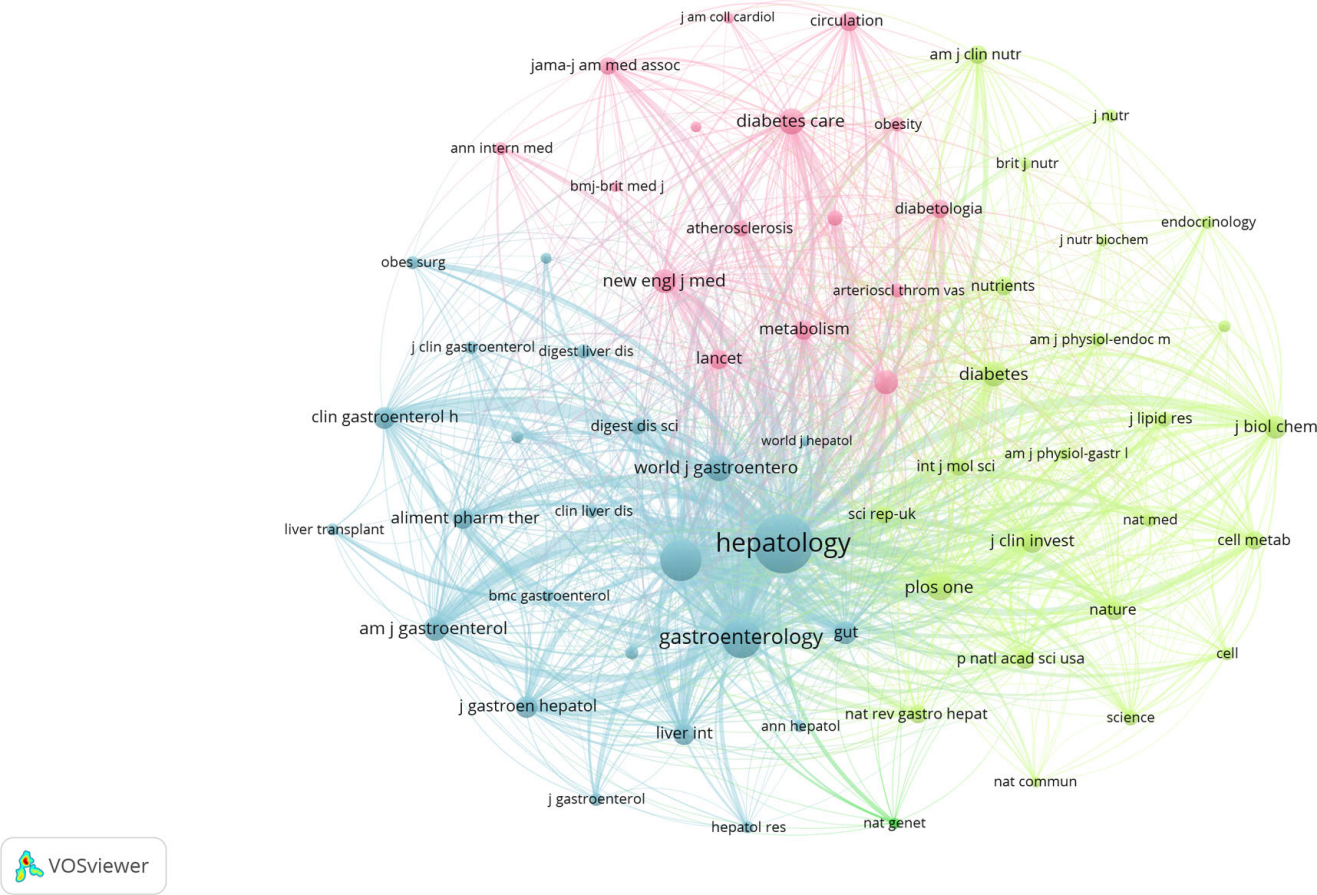
Journal co-citation network.

**Figure 7:**
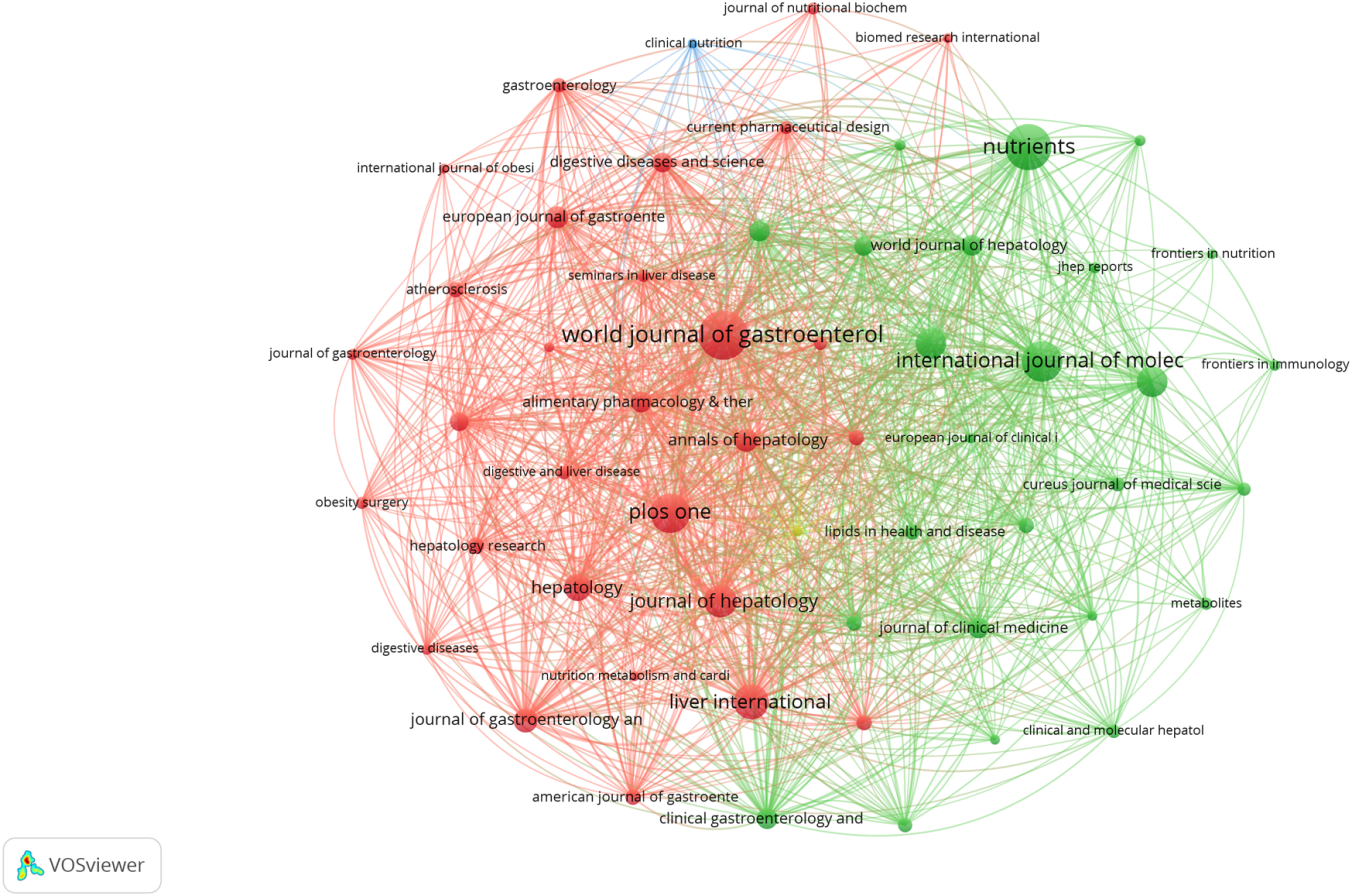
Bibliographic coupling networks of journals.

**Figure 8:**
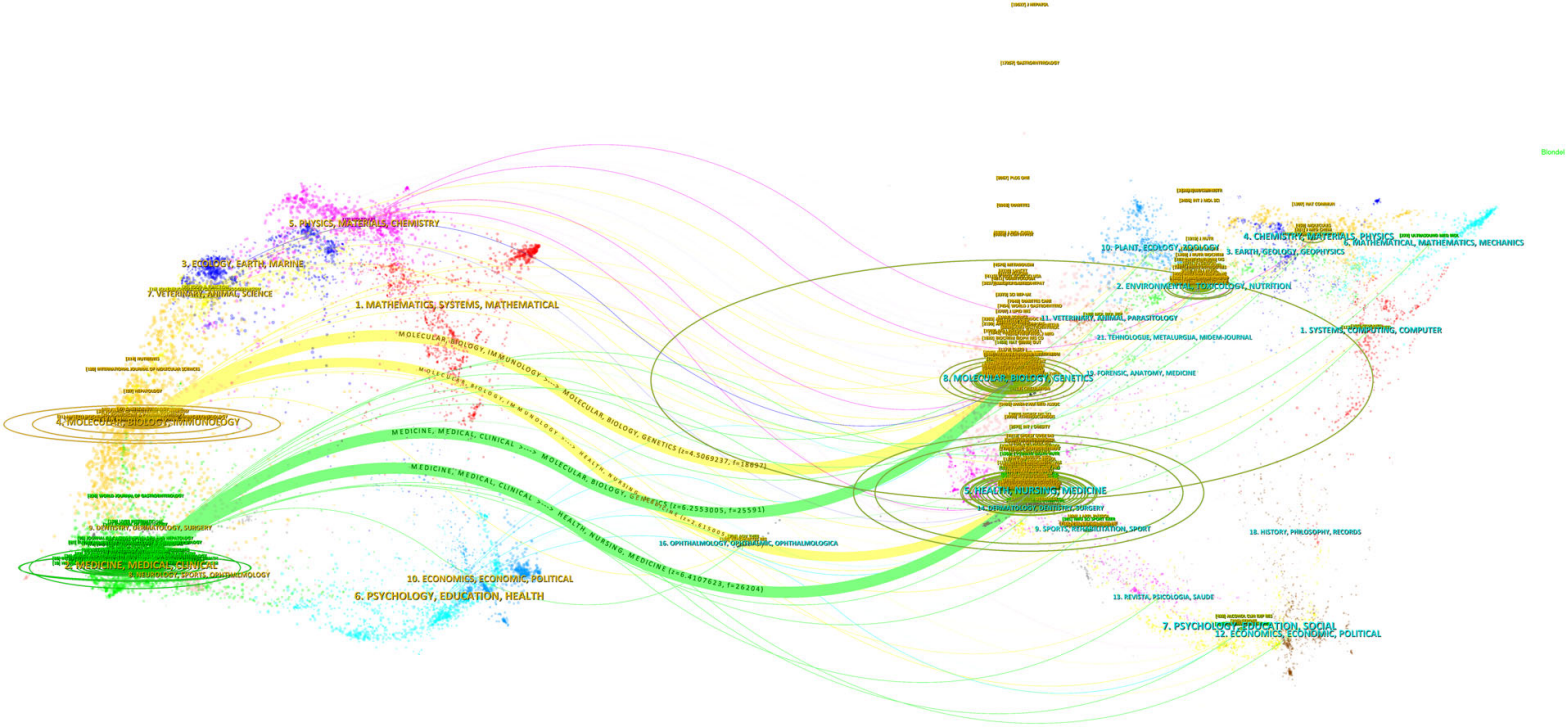
Dual-map overlay of journal publishing research.

Research on NAFLD and MetS was published across 1,872 academic journals, demonstrating a wide distribution. The *World Journal of Gastroenterology* led this field with 236 publications, accounting for 2.40% of the total, followed by *Nutrients* (214 publications, 2.18%) and the *International Journal of Molecular Sciences* (180 publications, 1.83%) (**Table 3**). Co-citation analysis identified the five core journals with the highest total link strength as follows: *Hepatology*, *Journal of Hepatology*, *Gastroenterology*, *PLOS ONE*, and *Diabetes* (**Figure 6**). Similarly, bibliographic coupling analysis revealed the five key journals with the strongest total link strength: *World Journal of Gastroenterology*, *International Journal of Molecular Sciences*, *Journal of Hepatology*, *Liver International*, and *Nutrients* (**Figure 7**). Furthermore, the dual-map overlay of journals (**Figure 8**) illustrated four primary citation paths (shown in yellow and green). These paths indicate that research published in journals categorized under *medicine/medical/clinical* and *molecule/biology/immunology* is predominantly cited by studies published in journals within the *health/nursing/medicine* and *molecule/biology/genetics* categories.

**Table 3:** Profile of leading publishing journals.

Source	Documents	Citations	Total link strength	IF
world journal of gastroenterology	236	15744	6157	5.4
nutrients	214	6933	2591	5.0
international journal of molecular sciences	180	7514	3316	4.9
plos one	178	6250	2327	2.6
liver international	150	7542	2710	5.2
journal of hepatology	141	27558	6907	33.0
frontiers in endocrinology	127	2338	1406	4.6
scientific reports	126	3515	1555	3.9
hepatology	107	32417	8057	16.8
journal of gastroenterology and hepatology	95	7700	2804	3.4

### 3.5 Analysis of authors and collaboration

**Figure 9:**
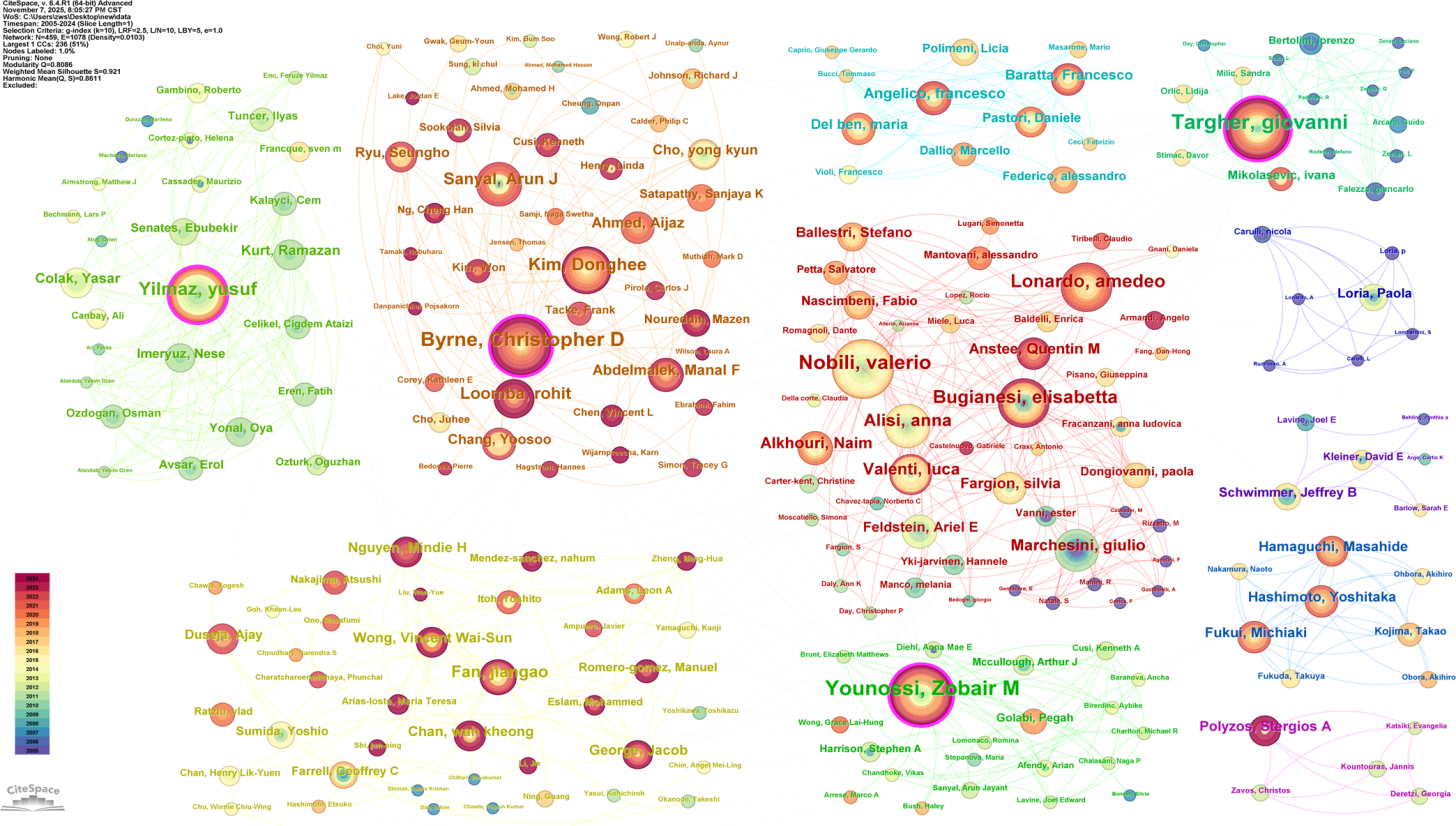
Visual network diagram of collaboration relationship among authors.

This study encompassed 9,821 publications authored by 44,299 individuals, indicating a broad author distribution and a moderate level of scholarly collaboration. To comprehensively understand the interactions and collaborations among authors, we adopted the g-index (k=10) node screening criterion in CiteSpace to visually analyze 459 authors. The resulting network illustrates the co-occurrence relationships and distribution patterns among core authors within the same research domain (**Figure 9**). The core authors were ranked in descending order of centrality (**Table 4**). Targher Giovanni emerged as the most influential contributor with the highest centrality, followed by Younossi Zobair M. and Yilmaz Yusuf.

**Table 4:** Publication and citation profiles of high-impact authors.

Author	Count	Centrality
Targher Giovanni	76	0.12
Younossi Zobair M.	74	0.12
Yilmaz Yusuf	48	0.11
Byrne Christopher D.	70	0.1
Bugianesi Elisabetta	44	0.09
Nobili Valerio	71	0.05
Lonardo Amedeo	42	0.05
Sanyal Arun J.	33	0.05
Loomba Rohit	28	0.05
Fan Jiangao	29	0.04

### 3.6 Reference analysis

**Figure 10:**
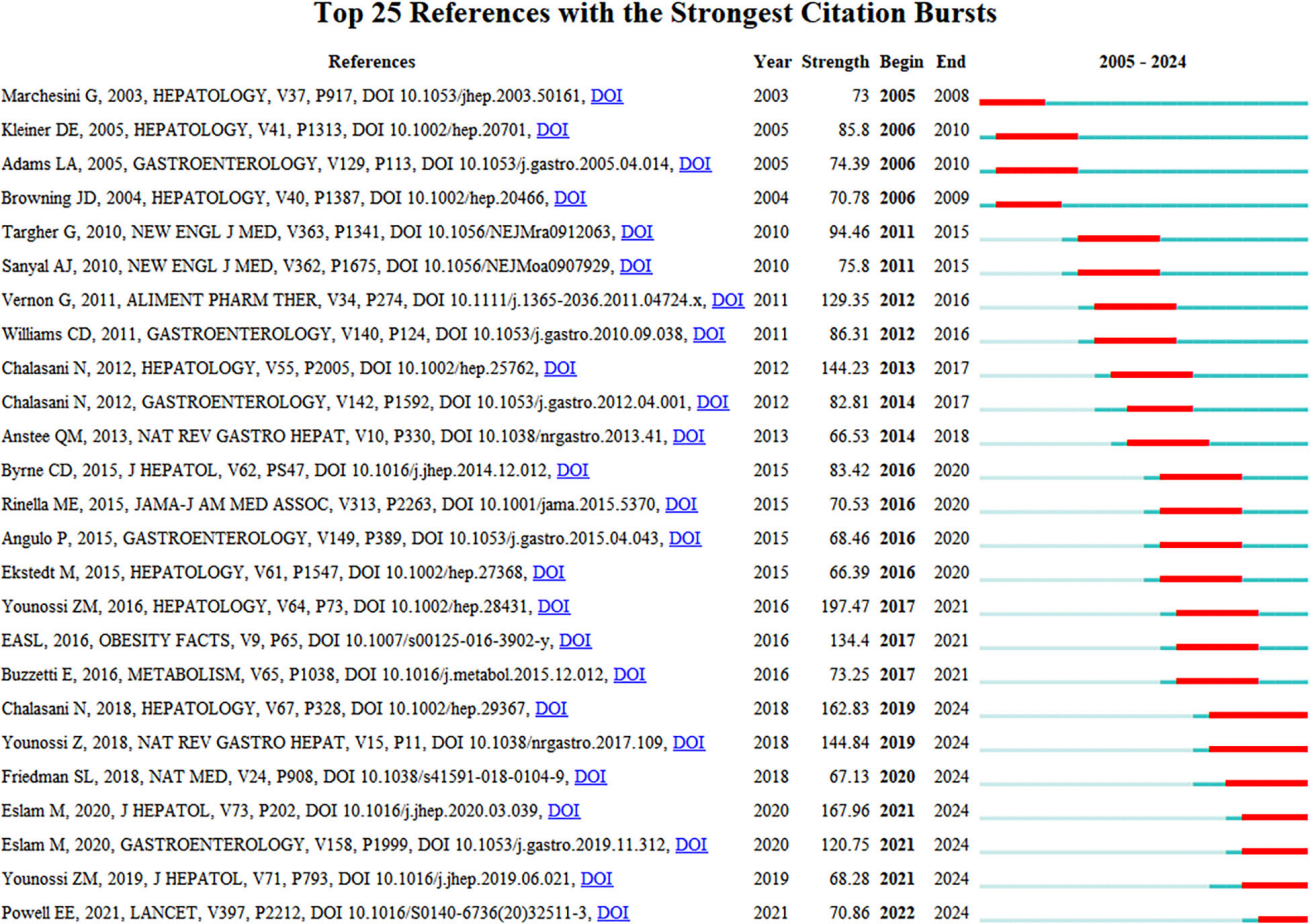
Top 25 most cited references.

**Figure 10** lists the top 25 most cited references. The dark blue line represents the time span from 2005 to 2024, while the red segment indicates the period of a citation burst, with a minimum burst duration set to 2 years. The most cited reference, which also possesses the strongest citation burst strength, is the article by Younossi Zobair M. et al., entitled “Global burden of NAFLD and NASH: trends, predictions, risk factors and prevention”, with a burst strength of 197.47 from 2021 to 2023. In recent years (2019-2024), seven references have exhibited sustained citation bursts. Among them, four demonstrated burst strengths exceeding 120. The reference with the highest burst strength during this period is the article by Eslam M. et al., entitled “MAFLD: a consensus-driven proposed nomenclature for metabolic associated fatty liver disease”, with a burst strength of 167.96.

### 3.7 Analysis of research hotspots and frontiers

**Figure 11:**
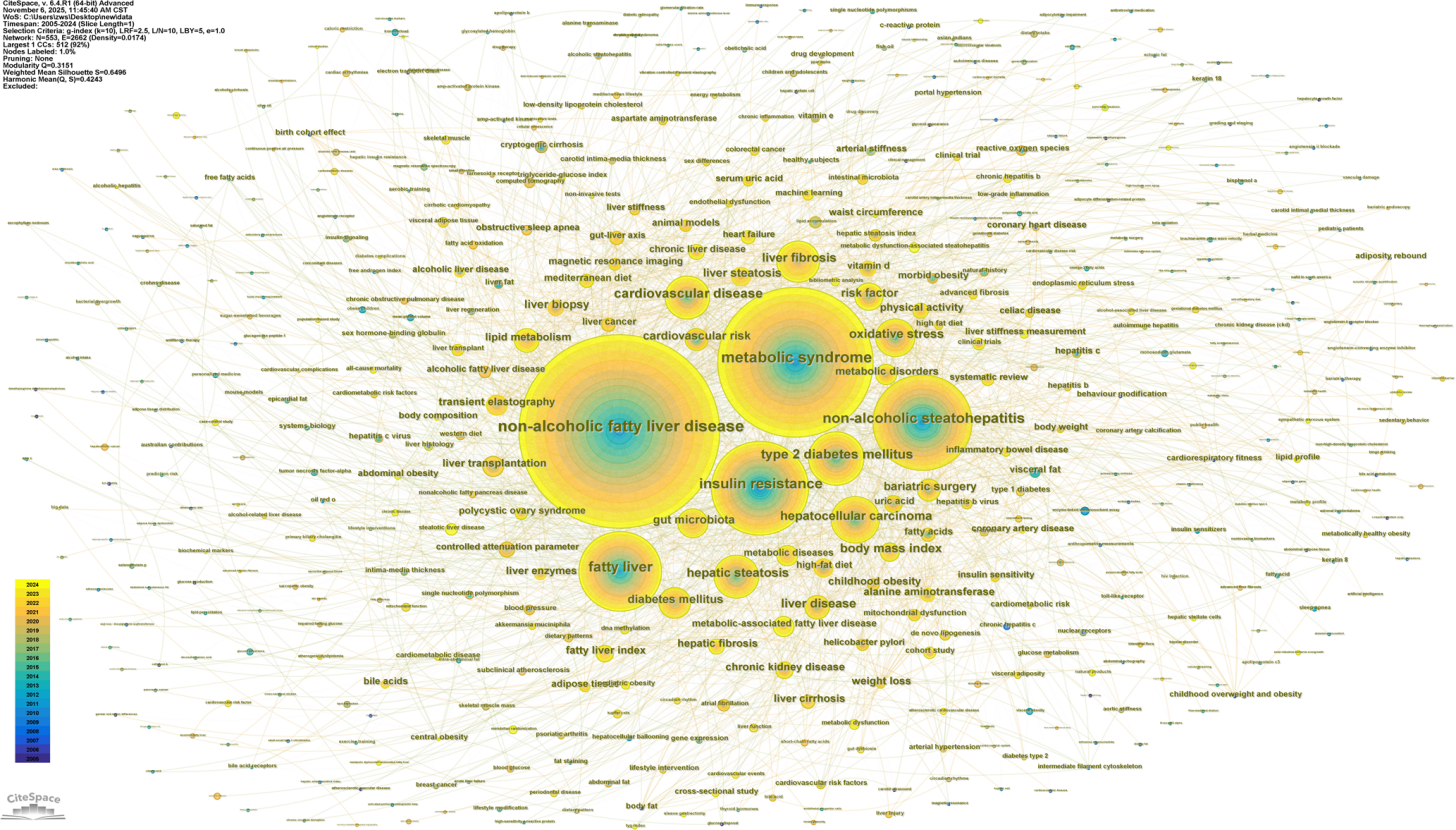
Keyword co-occurrence network.

**Figure 12:**
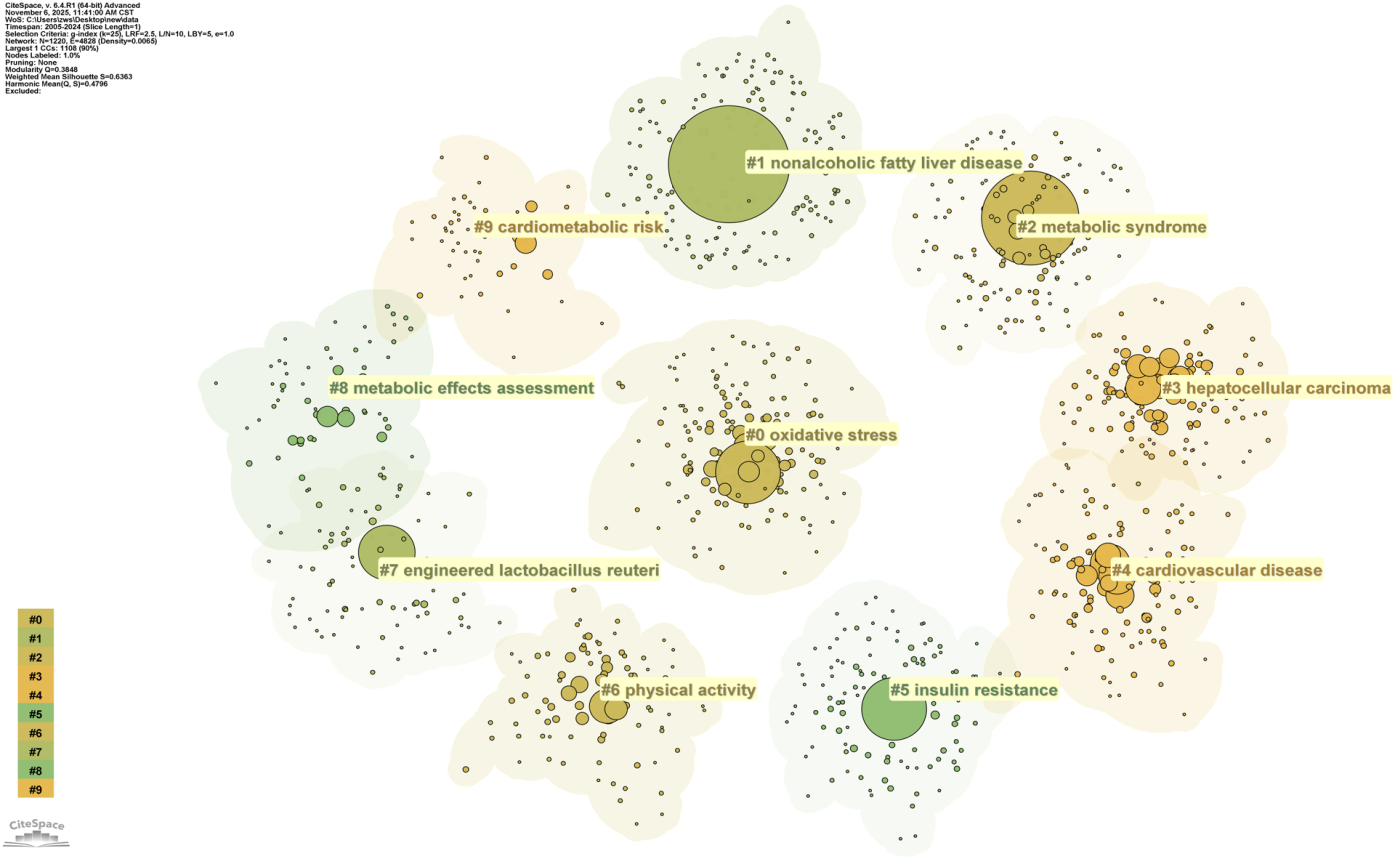
Keyword clustering analysis.

**Figure 13:**
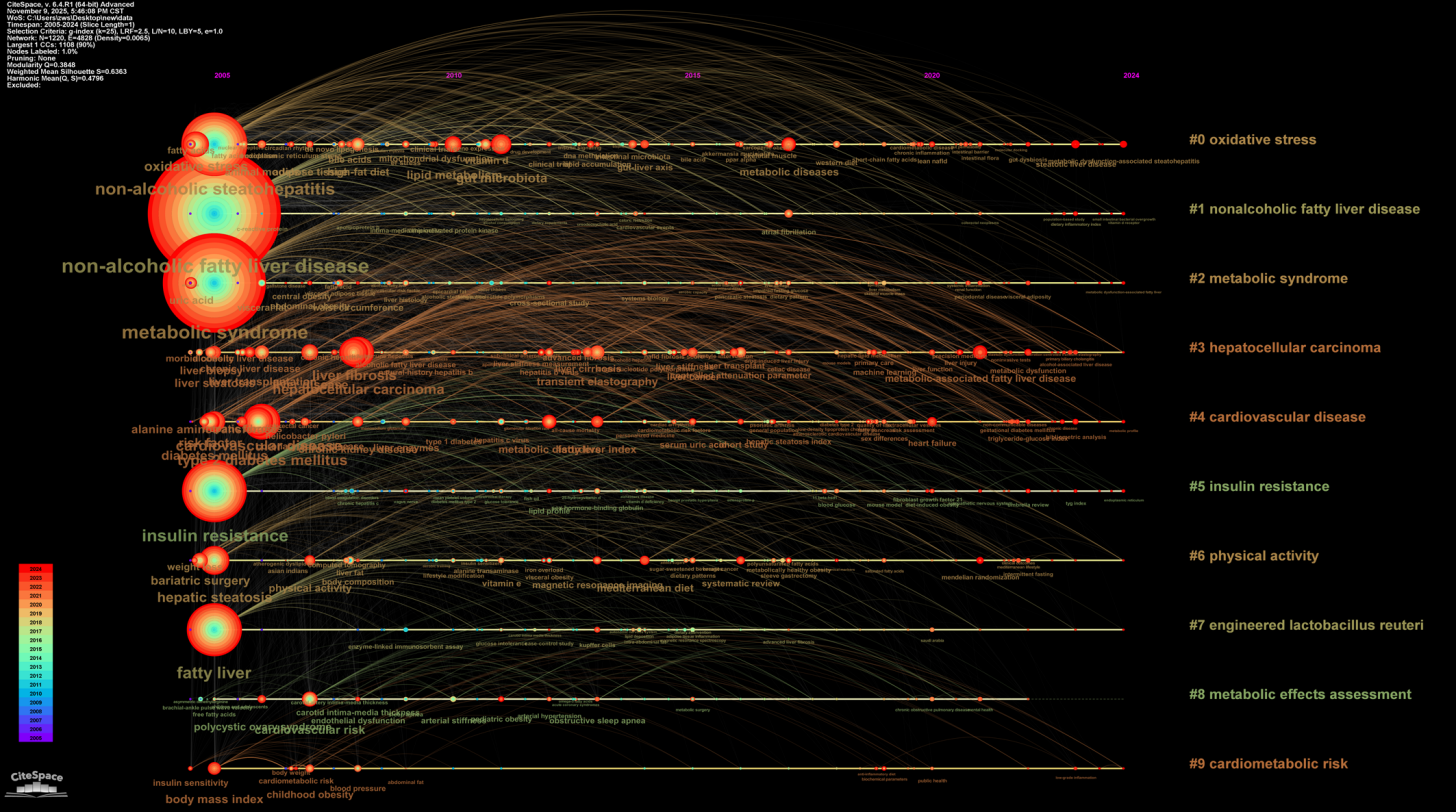
Keyword timeline map based on cluster analysis.

Keywords encapsulate the core concepts of publications and can clearly reveal research hotspots and frontiers within a discipline. A total of 6,262 keywords were identified in this study and subjected to a comprehensive analysis using CiteSpace. The parameters in CiteSpace were configured with a g-index (k=10) for node selection, while other settings remained at their defaults. The g-index evaluates the importance of keywords based on their cumulative occurrence frequency. This screening method sets a threshold according to frequency, where K=10 indicates a relatively high screening intensity, retaining only the keyword nodes with higher influence within each time slice. This yielded a keyword co-occurrence network (**Figure 11**), comprising 533 nodes where the size of each node is proportional to the keyword’s frequency of occurrence, effectively revealing the knowledge structure and emerging frontiers of the research field. The most frequent keywords were “non-alcoholic fatty liver disease” and “metabolic syndrome”, followed by “insulin resistance” and “non-alcoholic steatohepatitis”. **Figures 12** and **13** present the resulting 10 distinct clusters, which can be categorized into three primary research domains: First, pathological mechanisms related to MetS and MAFLD, represented by cluster #0 oxidative stress and #5 insulin resistance; second, disease progression pathways, encompassing cluster #1 nonalcoholic fatty liver disease, #2 metabolic syndrome, #3 hepatocellular carcinoma, and #4 cardiovascular disease; and finally, therapeutic approaches and assessment strategies, which include cluster #6 physical activity, #7 engineered lactobacillus reuteri, #8 metabolic effects assessment, and #9 cardiometabolic risk.

**Figure 14:**
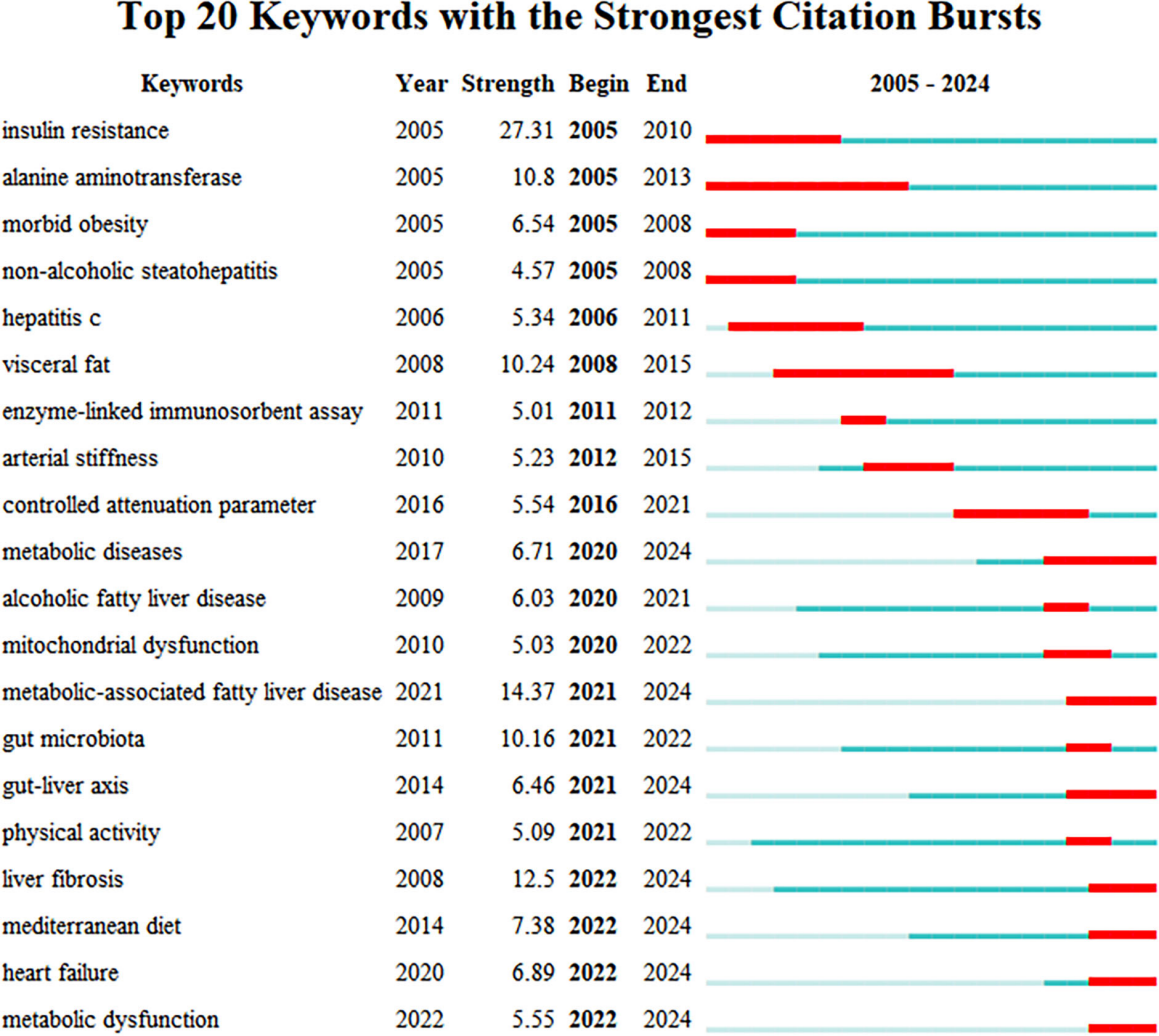
Top 20 keywords with the highest citation intensity.

**Figure 14** displays the top 20 keywords with the strongest citation bursts. In the early stage, research primarily concentrated on fundamental biological mechanisms and pathophysiological alterations. Keywords such as “insulin resistance”, “alanine aminotransferase”, “morbid obesity”, “non-alcoholic steatohepatitis”, and “visceral fat” reflected a focus on these core disease components and their underlying mechanisms. The research emphasis was on elucidating how metabolic disturbances initiate cellular injury and liver disease, highlighting the critical link between insulin resistance and hepatic health. As research progressed, the evolution of keywords indicated a deeper understanding of metabolic diseases. This phase saw increased attention to diagnostic tools and the complexity of pathological mechanisms. Keywords like “enzyme-linked immunosorbent assay”, “controlled attenuation parameter”, “metabolic diseases”, and “mitochondrial dysfunction” exemplified the exploration into the broader implications of metabolic dysregulation. Concurrently, the growing recognition of the connection between liver health and cardiovascular function was evidenced by increased research into “arterial stiffness” and metabolic status, leading to a more extensive and integrated research scope. In recent years, research has advanced towards more complex mechanisms, involving keywords such as “gut microbiota”, “gut-liver axis”, “physical activity”, “mediterranean diet”, “heart failure”, and “metabolic dysfunction”. The emergence of the new nomenclature “metabolic-associated fatty liver disease” itself signifies an evolved understanding of the disease’s pathogenesis and is anticipated to remain a central research focus. Furthermore, studies have increasingly focused on the role of gut microbes in metabolic regulation, underscoring the importance of the gut-liver axis in metabolic disorders. Simultaneously, research into lifestyle interventions, such as “physical activity” and the “mediterranean diet”, has gained significant traction. These developments reflect a deepening in strategies for preventing and managing metabolic diseases, marking a growing trend towards personalized medicine and early intervention.

## 4 Discussion

### 4.1 General characteristics of the included publications

This study conducted a systematic review of the literature on MetS and MAFLD published between 2005 and 2024.A total of 9,821 relevant and unique publications were identified and retrieved from the Web of Science Core Collection (WoSCC) and Scopus databases, forming a robust foundation for subsequent analysis. Over the past two decades, both publication output and citation frequency have demonstrated a consistent upward trajectory, with a particularly notable surge in scholarly output after 2020. This trend reflects a significant increase in research interest and solidifies the association between MetS and MAFLD as a central hotspot within the field.

### 4.2 Discussion on countries, institutions, journals and publications

In terms of publication volume, the United States, China, and Italy ranked as the top three contributing countries. The United States led in total output. Among the top five most prolific countries, the United States, Italy, and the United Kingdom all achieved average citation rates exceeding 70, indicating the substantial academic impact of their research. Although China’s publication count was comparable to that of the United States, its average citation frequency was considerably lower at 34.45. Although the number of papers published in China is similar to that in the United States, the average number of citations is only 34.45. This gap not only reflects the urgency of improving research quality, strengthening academic influence, and deepening cooperation among domestic scholars, but also highlights the need to account for differences between Eastern and Western academic traditions, linguistic environments, and research sample compositions. Therefore, while strengthening domestic academic networks, actively promoting international cooperation and fostering a more inclusive research ecosystem may become key strategies to enhance the global influence of China’s scientific output. Notably, the University of California System, Harvard University, and INSERM were the most prolific institutions, underscoring their leading role in advancing scholarship in this field. Regarding journals, *Hepatology*, *Journal of Hepatology*, and *World Journal of Gastroenterology* were the most frequently cited. *Hepatology* ranked first with over 30,000 citations, establishing itself as a pivotal force in this research domain. The three most-cited individual publications received 6,699, 4,061 and 3,151 citations, respectively. These seminal works were published in *Hepatology* (IF: 16.8), *Nature Reviews Gastroenterology & Hepatology* (IF: 51.6), and *Nature Medicine* (IF: 50.0) (31–33).

### 4.3 Temporal evolution of keywords

The evolving keyword trends over the past two decades reveal a clear trajectory in metabolic disease research: it has progressed from investigating fundamental biological mechanisms to conducting integrated studies on complex pathological processes and therapeutic strategies, forming a systematic research framework. The core keywords at each stage reflect the evolving understanding and conceptualization of both MetS and MAFLD.

In the early stage, research primarily focused on fundamental biological mechanisms and pathophysiological alterations. “Insulin resistance” was widely recognized as a central mechanism in the development of metabolic diseases, with numerous studies dedicated to elucidating its role in obesity and diabetes. “Alanine aminotransferase” served as a crucial biomarker of liver function, aiding researchers in monitoring hepatic injury and metabolic changes. Concurrently, the concept of “morbid obesity” highlighted the health risks associated with abnormal weight gain, particularly its close link to metabolic disorders. High levels of visceral fat were identified as a key factor influencing insulin sensitivity and overall health, directing attention to fat distribution and its metabolic implications. With the growing focus on obesity research, the term “glucagon-like peptide-1” has gained increasing prominence, and novel weight-loss drugs such as GLP-1 receptor agonists have also attracted considerable attention in the field. The focus of clinical intervention strategies is gradually shifting from traditional metabolic regulation toward targeted pharmacological approaches.

As research advanced, the focus expanded to encompass the complex mechanisms and clinical diagnosis of metabolic diseases. The introduction of techniques like the “enzyme-linked immunosorbent assay” facilitated the exploration of biomarkers, significantly enhancing the capacity to detect and monitor metabolic disturbances. Researchers also began to investigate “arterial stiffness” as an indicative marker linking cardiovascular and metabolic diseases, emphasizing the importance of cardiovascular health in overall metabolic management. As researchers’ attention to cardiovascular health-related keywords continues to grow, “SGLT2 inhibitors” have gradually emerged as a focal point in the field. As a class of novel glucose-lowering drugs, SGLT2 inhibitors have become an important emerging direction in metabolic disease research due to their advantages in cardiovascular protection. Simultaneously, “mitochondrial dysfunction” emerged as a research focus, with mitochondrial energy metabolism playing a central role in understanding metabolic dysregulation, particularly in obesity and diabetes. The deepening understanding of these complex mechanisms reinforced the recognition of the strong interconnection between MAFLD and MetS, driving the pressing need for effective treatments and interventions.

In recent years, research has increasingly shifted towards more complex disease mechanisms and multi-factorial integration. Studies on the “gut microbiota” and the “gut-liver axis” have proliferated, exploring the influence of gut microbes on metabolic regulation and their association with conditions like type 2 diabetes and MAFLD. Consequently, interventions targeting the gut microbiome have become a pivotal future direction. Simultaneously, the impact of lifestyle factors, such as “physical activity” and the “mediterranean diet”, has garnered increasing attention. This body of work underscores the importance and efficacy of lifestyle modifications in preventing and reversing metabolic diseases. Moderate physical activity and healthy dietary patterns can significantly improve metabolic parameters and reduce disease risk, thereby providing a crucial foundation for clinical intervention.

Collectively, research on metabolic diseases has undergone a significant evolution, moving from the exploration of basic mechanisms to interdisciplinary, multi-faceted investigation. The keywords at each stage mirror the shifting and deepening research priorities, demonstrating a progressively refined understanding of metabolic diseases. From a clinical perspective, this evolving trend suggests that the management of metabolic diseases has shifted from a therapeutic approach focused solely on glucose or lipid reduction toward a comprehensive model that emphasizes multisystem integration, cardiovascular protection, and lifestyle intervention. Future research directions are likely to place greater emphasis on personalized medicine, early intervention strategies, and the development of novel technological applications, aiming to achieve more substantial progress in the management of metabolic disorders. This evolutionary trajectory reflects the academic community’s progressively deeper exploration of issues related to MetS and MAFLD, showcasing multidimensional in-depth research and innovation within the field.

### 4.4 Shared pathogenic mechanisms between MetS and MAFLD

IR serves as a critical pathophysiological link between MAFLD and MetS, with both conditions exhibiting a significant bidirectional influence on the initiation and progression of IR (34). Insulin binds to receptors on the surface of target cells, activating downstream signaling pathways that regulate the metabolism of lipids, carbohydrates, and proteins. In MetS, IR leads to diminished cellular responsiveness to insulin, a phenomenon particularly evident in adipose tissue, skeletal muscle, and the liver. This impaired response triggers a cascade of metabolic disturbances that subsequently promote the development and progression of MAFLD (35). Under conditions of IR, the activation of key signaling pathways, notably the PI3K-Akt and MAPK pathways, is attenuated. This results in impaired glucose metabolism within hepatocytes and hindered glycogen synthesis (36). Concurrently, the diminished hepatic response to insulin exacerbates glycogen breakdown in the liver, contributing to elevated blood glucose levels (37).

Adipose tissue is not merely a passive energy reservoir but a highly active endocrine organ (38). Adipose tissue dysfunction is a hallmark of MetS and plays a pivotal regulatory role in the pathogenesis of MAFLD (39). Under metabolic dysregulation, the endocrine and immunomodulatory functions of adipose tissue undergo significant alterations (40). The expansion of adipose tissue, as seen in obesity, triggers a substantial increase in the secretion of pro-inflammatory factors, leading to a state of chronic low-grade inflammation. This inflammatory response is characterized notably by macrophage infiltration. Upon activation, these macrophages further release a cascade of additional inflammatory cytokines (41). These cytokines, in turn, induce systemic and hepatic insulin resistance while exacerbating hepatic inflammation, thereby aggravating IR and propelling the progression of both MAFLD and MetS (42).

Diets high in fat and sucrose can promote the accumulation of free fatty acids (FFA), thereby contributing to the development of hepatic disorders such as MAFLD (43, 44). Elevated FFA levels stimulate pancreatic β-cell proliferation and insulin secretion, leading to systemic hyperinsulinemia and a subsequent downregulation of insulin sensitivity (45). FFA not only triggers hepatic steatosis but also exacerbates the state of insulin resistance (46, 47). The combined effects of these factors—dysregulated adipokine secretion, immune cell infiltration, and increased FFA release—collectively establish a chronic pro-inflammatory state. This state not only accelerates the progression of MAFLD but also, by worsening insulin resistance and impairing glycemic control, contributes to the exacerbation of MetS (48).

Endoplasmic reticulum (ER) stress is closely associated with the pathogenesis and progression of MAFLD (49). An overload of nutrients can overwhelm the protein-folding capacity of the ER, a condition exacerbated by lipid accumulation. This triggers ER stress, characterized by the accumulation of misfolded or unfolded proteins within the ER lumen, which in turn activates the unfolded protein response (UPR) (50). ER stress, primarily through the UPR and associated inflammatory signaling, exacerbates hepatic lipid accumulation and insulin resistance. Concurrently, oxidative stress, driven by the accumulation of reactive oxygen species (ROS), contributes to hepatocyte injury and inflammatory responses. These stress pathways not only directly promote hepatic steatosis and fibrosis but also, by inducing systemic metabolic dysregulation and a low-grade inflammatory state, aggravate the clinical manifestations of MetS (51). Underlying factors such as adipose tissue dysfunction, insulin resistance, and gut microbiota dysbiosis act as both initiators and amplifiers of these stress responses, thereby establishing a self-reinforcing cycle that constitutes a shared pathological foundation for both MAFLD and MetS.

Oxidative stress represents a critical and indispensable factor in the pathogenesis of both conditions (52). It arises from an imbalance between the excessive production of reactive oxygen species (ROS) and the body’s antioxidant defenses, resulting in direct damage to cellular components such as lipids, proteins, and DNA (53). Studies indicate that in individuals with MetS, the interplay of obesity, insulin resistance, and inflammation synergistically elevates ROS levels, thereby exacerbating oxidative damage in the liver (54). In the progression of MAFLD, increased levels of free fatty acids (FFA), coupled with impaired mitochondrial β-oxidation, lead to excessive ROS accumulation. This not only triggers lipid peroxidation but also activates cell death pathways, promoting hepatocyte apoptosis and fibrosis (55). Oxidative stress is intricately linked to insulin resistance by impairing the insulin signaling cascade, which leads to reduced GLUT4 expression and disrupted glucose metabolism. This metabolic dysregulation, in turn, further aggravates hepatic steatosis, underscoring the complex and pivotal role of oxidative stress in the pathogenesis of both MetS and MAFLD (56).

Autophagy is a crucial homeostatic process that degrades unnecessary cellular components, thereby maintaining intracellular equilibrium. It plays a pivotal regulatory role in the pathophysiology of both MetS and MAFLD (57, 58). Dysregulation of autophagy contributes to hepatic lipid accumulation and exacerbates insulin resistance, which in turn can initiate the development of MetS and its associated complications (59). Under basal conditions, autophagy maintains hepatocyte functional integrity by selectively removing damaged organelles and misfolded proteins (60). In the progression of MAFLD, impaired autophagy not only aggravates lipid droplet accumulation but also induces hepatocyte injury and death. This leads to the release of damage-associated molecular patterns (DAMPs), which subsequently activate hepatic immune cells, thereby promoting inflammatory responses and driving fibrogenesis (61).

The composition of the gut microbiota plays a fundamental role in regulating host metabolism (62). Studies have revealed that gut dysbiosis is closely associated with the onset of MAFLD, whereas a healthy microbial composition aids in maintaining metabolic homeostasis, thereby influencing the development of both conditions (63). Gut dysbiosis not only disrupts energy harvest and metabolism but can also exacerbate insulin resistance by triggering systemic inflammation and promoting hepatic lipid deposition (64). Dysbiosis impairs intestinal barrier function and facilitates the translocation of endotoxins, thereby inducing hepatic endoplasmic reticulum stress and oxidative stress. Concurrently, hepatic metabolic disturbances further aggravate gut microbial imbalance, establishing a vicious cycle (65). This mutually reinforcing pathological interplay not only accelerates the progression of MAFLD but also serves as a significant driver for the development of MetS. Consequently, modulating the gut microbiota emerges as a promising therapeutic target for interrupting this self-perpetuating cycle of metabolic stress.

In recent years, novel mechanisms underlying the progression of MetS and MAFLD have emerged, among which the liver-spleen axis proposed by Italian researchers provides a new perspective for understanding the pathological mechanisms of both conditions (66). This axis is characterized by a bidirectional crosstalk where the spleen acts not just as a passive lymphoid organ, but as a central immune “sensor” and amplifier of systemic inflammation (67). The study suggests that chronic metabolic stress causes the spleen to integrate inflammatory cues, leading to the polarization of T-cells and the activation of macrophages that subsequently migrate to the liver to reinforce a profibrotic microenvironment. Furthermore, the researchers utilized spleen longitudinal diameter via ultrasonography as a non-invasive marker to evaluate this axis, correlating increased splenic volume with the severity of obesity-related hepatic inflammation (68). These findings strengthen the pathological connection between MetS and MAFLD, offering a promising target for mechanistic understanding and non-invasive clinical assessment.

### 4.5 Shared risk factors for MetS and MAFLD

Obesity, particularly visceral obesity, constitutes a key shared risk factor for both MAFLD and MetS. The pathological accumulation of visceral adipose tissue (VAT) plays a pivotal role in the pathogenesis and progression of both conditions (69). VAT is directly connected to the liver via the portal venous system, allowing free fatty acids (FFA) released from visceral adipocytes to rapidly reach the liver. This direct delivery promotes hepatic steatosis and subsequently exacerbates insulin resistance (70). Beyond mechanically increasing the hepatic lipid burden, VAT also promotes systemic low-grade inflammation and insulin resistance by secreting various adipocytokines (e.g., TNF-α, IL-6, leptin) and reducing adiponectin production (42, 71). A study comparing patient cohorts defined by body mass index (BMI) as obese (BMI ≥30 kg/m²) and lean (BMI <25 kg/m²) found no substantial difference in the prevalence of insulin resistance between the two groups (72). This observation suggests that body fat distribution, specifically the assessment of visceral fat, is more critical than relying solely on BMI measurement.

In patients with either MetS or MAFLD, the ability of insulin to suppress glucose production is impaired, leading to elevated blood glucose levels which, in turn, stimulate further insulin secretion (73). A meta-analysis revealed that individuals with MAFLD have a significantly elevated risk of progressing to type 2 diabetes mellitus and MetS over a 5-year follow-up period. This indicates that MAFLD is not merely a hepatic manifestation of metabolic aberration but also serves as an important independent predictor for the future development of metabolic diseases (74). Concurrently, a persistent state of hyperglycemia further exacerbates hepatic lipid accumulation. Hyperglycemia promotes the expression of key enzymes involved in the *de novo* lipogenesis pathway, thereby increasing hepatic fat synthesis and aggravating insulin resistance (75). Clinical studies demonstrate a markedly higher prevalence of MAFLD among diabetic patients, who also exhibit an accelerated rate of liver fibrosis progression compared to their non-diabetic counterparts. This evidence suggests that the interplay between hyperglycemia and MAFLD significantly aggravates the progression of liver disease (76).

Dyslipidemia is a prominent shared feature of both MetS and MAFLD, characterized particularly by hypertriglyceridemia and reduced levels of high-density lipoprotein cholesterol (HDL-C) (77). Furthermore, a bidirectional causal relationship exists between MetS and HDL dysfunction. On one hand, insulin resistance contributes to quantitative and qualitative abnormalities of HDL. On the other hand, functionally impaired HDL compromises insulin secretion, glucose uptake, and anti-inflammatory capacity, thereby exacerbating the underlying metabolic disturbances (78). Moreover, the dyslipidemic profile in MAFLD patients contributes to an increased risk of cardiovascular disease (79). Management of dyslipidemia not only helps alleviate the cardiovascular burden but can also confer benefits by ameliorating hepatic steatosis and inflammation to some extent (80).

Elevated blood pressure is included in the current diagnostic criteria for MetS, although the pathophysiological links are not fully elucidated. Early hypotheses proposed a mechanism initiated by central obesity and ectopic fat deposition, leading to insulin resistance and hyperinsulinemia. This, in turn, was thought to activate the sympathetic nervous system, promoting renal sodium retention and vasoconstriction, thereby elevating blood pressure (81). Recent studies suggest that hypertension in MetS is more likely mediated by mechanisms such as: physical compression of the kidneys by visceral fat, activation of the renin-angiotensin-aldosterone system (RAAS), and sympathetic nervous system (SNS) overactivity triggered by hyperleptinemia stimulating the hypothalamic melanocortin pathway (82). Concurrently, MAFLD is also significantly associated with hypertension. Studies indicate that hepatic fat accumulation increases the risk of hypertension independent of obesity, suggesting that MAFLD may contribute to elevated blood pressure through pathways involving chronic inflammation and dysregulated lipid metabolism (83, 84).

### 4.6 Limitations

It should be noted that this study has certain limitations. The literature data were sourced from only two databases and were restricted to English-language publications, which may not fully capture all relevant research in the field. The inclusion of review articles, while helpful for understanding the developmental trajectory of the field, may introduce certain biases in citation structure and hotspot analysis. The transition in terminology from NAFLD to MAFLD may lead to potential biases in the interpretation of data analysis, whereas inconsistencies in the standardization of institutional names may affect the accuracy of collaboration network analysis. Additionally, the inherent citation lag associated with bibliometric analysis may result in the underestimation of recently published high-quality research. The above factors collectively constitute the limitations of this study, yet they do not compromise the overall reliability of the conclusions to a significant extent. In future work, we will further expand our data sources and optimize keyword standardization to enhance the breadth and predictive accuracy of the research.

## 5 Conclusion

This bibliometric analysis provides a new reference and perspective for in-depth research on MetS and MAFLD. The marked increase in publications from 2005 to 2024 underscores the growing global academic importance of this field. The United States has emerged as the leading contributor, followed by China and Italy. Enhancing global collaboration is crucial for fostering broader research initiatives. The University of California System stands as the most prolific institution, while esteemed journals such as *Hepatology*, *Journal of Hepatology*, and *World Journal of Gastroenterology* play a pivotal role in advancing the discipline. Younossi Zobair M. received the highest number of citations, whereas Targher Giovanni ranked highest in centrality. The evolution of keyword trends reveals a shift from early focuses on pathophysiological foundations such as “insulin resistance” and “morbid obesity” to emerging terminology including “mitochondrial dysfunction” and “controlled attenuation parameter.” In recent years, research frontiers have advanced towards “gut microbiota,” the “gut-liver axis,” and lifestyle interventions. This evolutionary trajectory signifies a profound paradigm shift within the field, moving from traditional pathophysiological description towards mechanism-based precise diagnosis and early intervention strategies. In summary, this study depicts the research structure and evolving trends in the field of MetS and MAFLD through bibliometric analysis. The findings may help researchers identify emerging trends and research hotspots in this field, and provide references for seeking potential collaborators and suitable journals, thereby contributing to the continuous advancement of research in this area.

## Data Availability

The raw data supporting the conclusions of this article will be made available by the authors, without undue reservation.
